# Form of Supplemental Selenium Affects the Expression of mRNA Transcripts Encoding Selenoproteins, and Proteins Regulating Cholesterol Uptake, in the Corpus Luteum of Grazing Beef Cows

**DOI:** 10.3390/ani12030313

**Published:** 2022-01-27

**Authors:** Sarah N. Carr, Benjamin R. Crites, Joy L. Pate, Camilla H. K. Hughes, James C. Matthews, Phillip J. Bridges

**Affiliations:** 1 Department of Animal and Food Sciences, University of Kentucky, Lexington, KY 40546, USA; sarah.carr@uky.edu (S.N.C.); benjamin.crites@uky.edu (B.R.C.); james.matthews@uky.edu (J.C.M.); 2 Department of Animal Sciences, Center for Reproductive Biology and Health, The Pennsylvania State University, University Park, PA 16802, USA; jlp36@psu.edu (J.L.P.); camilla.hughes@umontreal.ca (C.H.K.H.)

**Keywords:** corpus luteum, selenium, selenoprotein, progesterone, cholesterol, steroidogenesis

## Abstract

**Simple Summary:**

In regions with selenium (Se) deficient soils, producers should supplement this mineral to the diet of forage-grazing cattle. Commercial supplements are typically formulated using inorganic forms of Se, but the organic forms are those naturally available in forages. We previously reported that circulating concentrations of progesterone are affected by the form of Se supplemented to cows. In this report, we aimed to determine (1) how the form of Se affects the expression of mRNA encoding selenoproteins in the corpus luteum, and (2) whether form of Se-induced increases in progesterone are the result of direct increases in the expression of steroidogenic transcripts. Cows were supplemented with the industry standard, an inorganic form of Se, or a 1:1 mix of organic and inorganic forms (MIX), with corpora lutea recovered on Day 7 of the estrous cycle. Transcripts encoding selenoproteins, as well as those regulating the uptake of cholesterol were affected by form of Se, but not those directly regulating steroidogenesis. We demonstrated that the expression of multiple selenoprotein mRNAs are affected by the form of Se, some of which are associated with intracellular antioxidant activity, and that the previously reported form of Se-induced increase in progesterone appears to be due to increased cholesterol availability by the corpus luteum, not by an increase in the expression of key steroidogenic enzymes.

**Abstract:**

Selenium (Se)-deficient soils necessitate supplementation of this mineral to the diet of forage-grazing cattle. Functionally, Se is incorporated into selenoproteins, some of which function as important antioxidants. We have previously shown that the source of supplemental Se; inorganic (sodium selenite or sodium selenate; ISe), organic (selenomethionine or selenocysteine; OSe) or 1:1 mix of ISe and OSe (MIX), provided to Angus-cross cows affects concentrations of progesterone (P4) during the early luteal phase of the estrous cycle. In this study, we sought to investigate (1) the effect of form of Se on the expression of mRNA encoding selenoproteins in the corpus luteum (CL), and (2) whether this previously reported MIX-induced increase in P4 is the result of increased luteal expression of key steroidogenic transcripts. Following a Se depletion and repletion regimen, 3-year-old, non-lactating, Angus- cross cows were supplemented with either ISe as the industry standard, or MIX for at least 90 days, with the CL then retrieved on Day 7 post-estrus. Half of each CL was used for analysis of targeted mRNA transcripts and the remainder was dissociated for culture with select agonists. The expression of three selenoprotein transcripts and one selenoprotein P receptor was increased (*p* < 0.05), with an additional five transcripts tending to be increased (*p* < 0.10), in cows supplemented with MIX versus ISe. In cultures of luteal cells, hCG-induced increases in P4 (*p* < 0.05) were observed in CL obtained from ISe-supplemented cows. The abundance of steroidogenic transcripts in the CL was not affected by the form of Se, however, the abundance of mRNA encoding 2 key transcripts regulating cholesterol availability (*Ldlr* and *Hsl*) was increased (*p* < 0.05) in MIX-supplemented cows. Overall, the form of Se provided to cows is reported to affect the expression of mRNA encoding several selenoproteins in the CL, and that the form of Se-induced effects on luteal production of P4 appears to be the result of changes in cholesterol availability rather than a direct effect on the expression of steroidogenic enzymes within the CL.

## 1. Introduction

The physiological effects of a deficiency in selenium (Se) that occur as a result of cattle consuming forages from Se-deficient soils are well-documented [1,2,3,4,5]. In Se-deficient regions, including the southeast United States, it is recommended that cattle producers supplement this trace mineral to ensure adequate rates of growth [1], immune function [2,3], and reproductive health [4,5]. Naturally, Se can exist in various forms, including the organic forms, selenocysteine (SeCys) and selenomethionine (SeMet), and the inorganic forms, selenite and selenate [6]. Organic forms of selenium are the most predominant form in forages; however, inorganic forms are more commonly used in commercial vitamin-mineral mixes supplemented to livestock in selenium deficient regions such as the southeast United States [7].

The relevance of Se as a trace mineral was identified due to its role as a structural component of glutathione peroxidases (GPX’s; [8]), a family of enzymes with antioxidant capabilities. Presently, there have been >20 genes for selenoproteins identified in humans [9] and pigs [10]. Many studies have demonstrated the antioxidant capabilities of selenoproteins to protect cells from harmful reactive oxygen species (ROS) [9,11,12,13,14,15,16,17,18,19]. GPX variants are some of the most common antioxidants that catalyze hydrogen peroxide into H_2_O [9], and thioredoxins protect against oxidative stress as redox proteins and can catalyze dithiol-disulfide exchange reactions in various tissues. Although thioredoxin is not a selenoprotein, once thioredoxin is oxidized, it must be reduced by the selenoproteins thioredoxin reductases (TRXR) [11]. Additionally, selenoprotein P (SELENOP [14]), selenoprotein W (SELENOW [15]), selenoprotein H (SELENOH [16]), selenoprotein K (SELENOK [17]), selenoprotein M (SELENOM [18]), and selenoprotein R (SELENOR [19]) have documented or proposed antioxidant properties.

Physiological incorporation of Se is dependent on its respective form [6] and the form of Se available for uptake affects the transcriptome profile in tissues including the liver [20], neonatal testis [21], and pituitary [22] of cattle. The form of dietary Se affects the bioavailability and bioactivity of this trace mineral in blood and tissues, altering concentrations of selenoproteins including GPX’s [23,24,25]. Research has indicated differences in the assimilation of Se, dependent on the form being fed, with lower blood and milk concentrations of Se in cows supplemented with an inorganic (ISe) versus organic (OSe) form [25,26,27,28,29,30,31,32]. Previously, our lab demonstrated that a 1:1 blend of ISe and OSe (MIX) provided to cows to achieve a Se-adequate status resulted in a 1.0 to 1.7 ng/mL increase in early (Days 6 and 7) luteal phase concentrations of P4 when compared to cows supplemented with ISe and/or OSe alone [33,34]. This elevated P4 occurs without any effect on diameter of the CL [33]. Importantly, multiple trials have reported that development of the endometrium [35] and consequently growth of the conceptus [36,37] are advanced with elevated early luteal phase concentrations of P4, increasing overall indicators of fertility [38].

With no knowledge of how different forms of supplemental Se affect the expression of transcripts encoding selenoproteins in the CL, nor of the mechanism by which supplementation with MIX increases early luteal phase [33,34] or gestational [34] concentrations of systemic P4, the primary objectives of this study were to determine the effect of form of Se on (1) the relative abundance of mRNA transcripts that encode selenoproteins and targeted steroidogenic enzymes in the CL, and (2) the ability of dissociated luteal cells to synthesize P4 and respond to key agonists in vitro. To achieve this goal, we assigned cows to form of Se-treatment regimens (ISe versus MIX) that achieved a Se-adequate status in all cows, then collected CL from these cows on Day 7 of the estrous cycle for molecular and in vitro analyses. We hypothesized that there would be form of Se-induced changes in the expression of selenoprotein mRNAs, that CL from MIX-treated cows would express an increase in enzymatic transcripts that favor the production of P4 by luteal cells, and an increased ability for these luteal cells to respond to exogenous agonists in vitro.

## 2. Materials and Methods

### 2.1. Animals and Experimental Procedure

**Figure 1:**
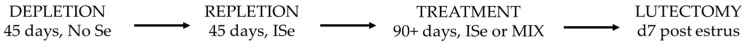
Experimental model of dietary supplementation. At the start of experimentation, cows were subject to a 45-day depletion during which they were supplemented with a Se-free vitamin-mineral mix, followed by a 45-day repletion period during which cows were supplemented with a vitamin-mineral mix formulated to contain 35 ppm ISe/cow/day. After repletion, cows were supplemented with a vitamin-mineral mix containing 35 ppm of their respective treatment for at least 90 days prior to estrous synchronization with an i.m. injection of 25 mg dinoprost tromethamine. On day 7 post-estrus, CL were collected via trans-vaginal lutectomy for analysis of key selenoprotein-encoding and steroidogenic transcripts by real-time PCR and in vitro culture of dissociated luteal cells with select agonists.

All procedures involving animals were approved by the University of Kentucky’s Institutional Animal Care and Use Committee (IACUC), protocol number 2017-2828. Fall-born, first-calf 3-year-old Angus-cross cows (N = 10) were selected from established, Se form-specific cowherds as previously described [20,21,26,33]. At the beginning of this trial, all cows were subject to a 45-day period where systemic levels of Se were depleted (supplementation of a vitamin-mineral mix containing no exogenous source of Se), followed by a 45-day period where all cows received supplement formulated with 35 ppm Se as ISe (repletion, Figure 1) to return total blood Se in all cows to a Se adequate status [24,39,40]. Following this, cows were assigned to at least 90 days of individual access to ISe (*n* = 5; Sodium selenite; Prince Agri Products, Inc., Quincy, IL, USA) or MIX (*n* = 5; 1:1 ISe:OSe; SEL-PLEX; Alltech, Inc., Nicholasville, KY, USA). Cows were individually supplemented with their respective treatments using in-pasture Calan gates [26]. Jugular blood was collected for the determination of total whole blood Se from each cow at the start, middle and endpoint of the depletion and repletion periods, then monthly until the end of the trial, similar to methods previously described [26,33].

### 2.2. Diet

Cows were initially maintained on a common, nontoxic endophyte-infected tall fescue pasture (Lacefield MaxQ II), then transferred to a common silage diet during the winter months (January–April). Forage samples for analysis of Se and trace minerals were collected for the duration of experimentation. Pasture concentration of Se in the forage was approximately 0.01 mg Se/kg as fed and 0.04 mg Se/kg dry matter (Dairy One Forage Testing Laboratory, Ithaca, NY, USA). The concentration of Se in the dietary corn silage was approximately 0.03 mg Se/kg as fed and 0.08 mg Se/kg dry matter (Dairy One Forage Testing Laboratory, Ithaca, NY, USA). Both are consistent with being classified as Se deficient [41,42].

### 2.3. Experimental Regimen

Following the 90 days of treatment with supplemental Se in either ISe or MIX forms, cows were randomly treated i.m. with 25 mg dinoprost tromethamine (PGF_2α_; Lutalyse, Pfizer Animal Health, New York, NY, USA) to induce luteal regression and monitored for behavioral estrus (Day 0). Development of the preovulatory follicle and ovulation was confirmed via trans-rectal ultrasonography using a 5–8 MHz, 66-mm linear array transducer (Ibex Pro, E.I. Medical Imaging, Loveland, CO, USA). On days 5, 6, and 7 post-estrus, the diameter of the CL was determined by ultrasonography and blood (8 mL) was collected into additive-free tubes (Vacutainer, Becton, Dickinson and Company, Franklin Lakes, NJ, USA) for retrieval of serum and quantification of P4 via radioimmunoassay [43]. On Day 7, each CL was collected by supra-vaginal lutectomy, and immediately placed in ice-cold culture medium (24 mM HEPES-buffered Ham’s F-12 (1×) culture medium plus L-glutamine and sodium bicarbonate containing 0.5% bovine serum albumin and 20 µg/mL gentamicin) to be washed, weighed, and cut into two halves [44,45]. One-half of the CL was transported in the culture medium on ice to the laboratory for dissociation and culture of the fully differentiated luteal cells. The second half was divided into 8 pieces, each of which was immediately snap frozen in liquid nitrogen to be used for RNA extraction and quantitative transcript expression by real-time polymerase chain reaction (qPCR).

### 2.4. Cell Culture

Luteal tissue was handled, dissociated, and incubated following established protocol [44,45]. Briefly, luteal tissue was minced into ~1 mm^3^ cubes and placed in 24 mM HEPES-buffered Ham’s F-12 (1×) culture medium with L-glutamine and sodium bicarbonate (Gibco^®^, Life Technologies Corporation™, Grand Island, NY, USA), containing 0.5% BSA (Fisher BioReagents, Fair Lawn, NJ, USA), 20 µg/mL gentamicin (Gibco^®^, Life Technologies Corporation™, Grand Island, NY, USA), and 2000 U/g tissue collagenase type I (Worthington Biochemical Corporation, Lakewood, NJ, USA) to dissociate cells. Following dissociation, cells were re-suspended in Ham’s F-12 solution with L-glutamine, sodium bicarbonate, HEPES buffer and gentamicin for the determination of cell numbers and viability.

Culture plates were prepared by coating each well in 250 µL culture medium with 10% newborn calf serum (Gibco^®^, Life Technologies Corporation™, Grand Island, NY, USA). Plates were incubated for 30 min and then washed with the serum-free medium. Luteal cells were plated at 0.6 × 10^6^ cells/mL in 24-well plates in serum free medium supplemented with L-glutamine and ITS (5 µg/mL insulin, 5 µg/mL transferrin, and 5 ng/mL selenium, Corning™ Premix Universal Culture Supplement, Corning, NY, USA); and 20 µg/mL gentamicin. Steroidogenic cells were cultured with or without PGE_2_ (1 and 50 µM/mL, Cayman Chemical Company, Ann Arbor, MI, USA), LH (1 and 10 IU/mL, NIH LH-S26), or hCG (10 and 50 ng/mL, Sigma Aldrich, St. Louis, MO, USA). Treatment concentrations (low and high for each agonist) were based upon previous reports [46,47,48,49,50,51,52]. All treatments and agonist-free control groups were performed in triplicate wells within each replicate. Cultures were incubated at 37 °C in a 5% CO_2_/95% air environment. Cultures were observed every 12 h during experimentation. All cultured wells were similarly confluent and did not display any loss of cells. Culture media were collected and replaced with fresh medium containing treatments every 24 h for a total of 96 h for subsequent determination of the concentration of P4 via RIA.

### 2.5. Real-Time PCR

Total RNA was extracted from 400–600 mg frozen luteal tissue using TRIzol Reagent (Invitrogen Corporation, Carlsbad, CA, USA) following the manufacturer’s instructions. The purity and concentration of total RNA samples were analyzed using a NanoDrop ND-100 Spectrometer (Nanodrop Technologies, Wilmington, DE, USA). All samples had 260/280 absorbance ratios of 1.97 or greater.

The quantification of relative mRNA was performed using real-time polymerase chain reaction (qPCR) using cDNA. To do so, 1 μg of each cow’s total RNA was reverse transcribed into cDNA using the SuperScript™ IV VILO™ Master Mix with ezDNAse™ Enzyme (Invitrogen by Thermo Fisher Scientific, Vilnius, Lithuania). Additionally, a control reaction that did not contain reverse transcriptase was performed and analyzed via qPCR to ensure that products from the targeted transcripts were not obtained from genomic DNA contamination.

The relative abundance of the following mRNA’s was quantified: the iodothyronine deiodinases *Dio1*, *Dio2*, and *Dio3*, the glutathione peroxidases *Gpx1*, *Gpx2*, *Gpx3*, *Gpx4*, and *Gpx6*, the thioredoxin reductases *Txnrd1*, *Txnrd2*, and *Txnrd3*. Additionally, we investigated the following selenoprotein mRNA’s: *Selenof*, *Selenoh*, *Selenoi*, *Selenok*, *Selenom*, *Selenon*, *Selenoo*, *Selenop*, *Selenor*, *Selenos*, *Selenot*, *Selenov*, *Selenow*, *Sephs1*, and *Sephs2*. Finally, we analyzed the selenoprotein P receptor mRNA’s *Lrp2*, *Lrp8*, and *Tfrc*.

Next, the relative abundance of P4-associated enzymatic transcripts: *Star*, *Cyp11a1*, *Hsd3b1*, *Ptgs2*, and *Ptges*, the receptor transcripts: *Lhcgr*, *Pgr*, *Pgrmc1*, *Pgrmc2*, *Paqr5*, *Paqr7*, *Paqr8*, *Ep1*, *Ep2*, *Ep3*, *Ep4*, and *Pgtfr*, and transcripts of proteins associated with cholesterol availability: *Ldl*, *Scarb1*, *Hsl*, *Npc1*, and *Npc2* were quantified. Primer sequences used in qPCR and GenBank accession numbers are listed in Tables S1 and S2. Real-time PCR procedures were performed using the Bio-Rad CFX Maestro™ thermal cycler (Bio-Rad, Hercules, CA, USA) with iTaq Universal SYBR^®^ Green Supermix (Bio-RAD, Hercules, CA, USA). A total volume of 25 μL was used in each qPCR reaction containing 5 μL of cDNA, 1 μL of a 10 μM stock of each primer (forward and reverse), 12.5 μL of 2X SYBR Green PCR Master Mix, and 5.5 μL of nuclease-free water. The relative amount of each transcript was calculated using the 2^−ΔΔCT^ method [53]. Primer sets for genes of interest were designed and obtained from NCBI Primer-BLAST tool (https://www.ncbi.nlm.nih.gov/tools/primer-blast/, accessed 12 October 2021) against RefSeq sequence.

All cDNA products were validated via DNA sequencing for verification of targets at Eurofins MWG Operon LLC (Louisville, KY, USA), as previously described [21,54]. The resulting sequences were then compared to the NCBI RefSeq mRNA sequences used for primer templates. The primer pair design, amplicon length of product, and product identity for each targeted transcript are shown in Tables S1 and S2. Three constitutively expressed genes (*β-actin*, *Hprt1*, and *Sdha*) with CT values not affected (*p* > 0.05) by Se-form treatment were used to normalize the relative mRNA expression to the geometric mean of these three genes. For qPCR analysis, *n* = 5 and 5 for ISe and MIX treatments, respectively. All reactions were performed in triplicate.

### 2.6. Se and P4 Analysis

Total blood Se was assayed by the American Associates for Veterinary Laboratory Diagnosticians-approved University of Kentucky Veterinary Diagnostics Laboratory (Lexington, KY, USA), as previously reported [55].

In both samples of serum and in vitro media, concentrations of P4 were quantified by a commercially available competitive RIA without extraction (ImmuChem™ Coated Tube Progesterone 125-I RIA Kit, MP Biomedicals, Costa Mesa, CA, USA), as described previously [31]. There was one assay performed for analysis of the serum with an intra-assay CV of 5%, and there were seven assays performed for analysis of culture media with inter-assay CV of 7.85% and intra-assay CVs ranging from 4.53–9.43%.

### 2.7. Statistical Analysis

In all statistical analyses, an individual cow was the experimental unit. All data were analyzed for a normal distribution and homogeneity of variance. When appropriate, data were natural log-transformed for normality. Results are presented as Least Square Means (LS Means) ± standard error of the mean (SEM). At *p* < 0.05 significance was declared, with a tendency to differ when 0.05 ≤ *p* < 0.10. The effect of treatment on concentrations of peripheral Se and P4 were analyzed as an ANOVA with repeated measures using the PROC MIXED function of SAS statistical software package (version 9.4; SAS Institute, Inc., Cary, NC, USA). The form of dietary Se was considered the fixed effect for both, and the P4 data were natural log-transformed due to not being normally distributed. Luteal weight, luteal diameter, and relative abundance of all mRNA transcripts were analyzed using a Student’s *t*-Test with the PROC TTest function of SAS statistical software package (version 9.4; SAS Institute, Inc., Cary, NC, USA). In vitro concentrations of P4 were analyzed using a split plot design for repeated measures (culture time) with Se treatment as the main-plot and LH, PGE_2_, and hCG as the sub-plot factors. Data were blocked by CL (cow) and natural log transformed due to data not being normally distributed or homologous. Data were analyzed as a mixed ANOVA using the PROC GLIMMIX function of the SAS statistical software package (version 9.4; SAS Institute, Inc., Cary, NC, USA).

## 3. Results

### 3.1. Concentrations of Se in Whole Blood

**Figure 2:**
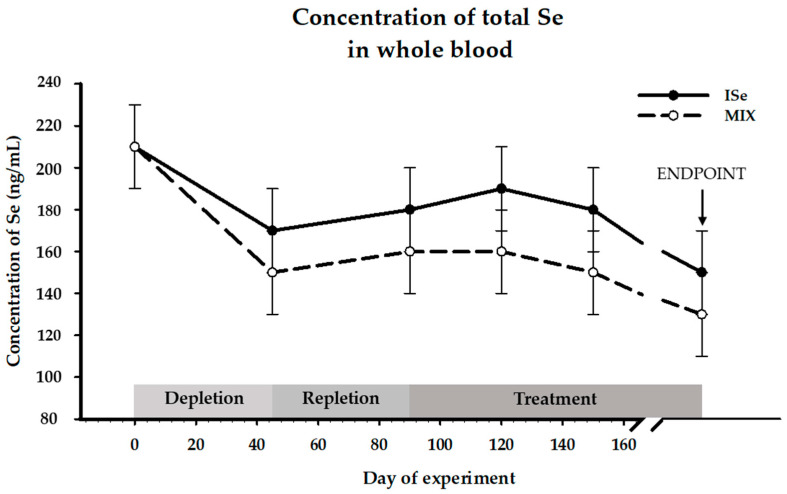
Effect of treatment (form of Se) on concentrations of Se in whole blood (ppm; LS Mean ± SEM) of Se in cows supplemented with either ISe (Sodium selenite; *n* = 5) or a 1:1 combination (MIX) of ISe and OSe (Sel-Plex; *n* = 5). Data were analyzed as an ANOVA with repeated measures. Whole blood Se was not affected by treatment (*p* = 0.2393) but was affected by time (*p* < 0.0001).

All cows were maintained on their respective Se-form treatments (ISe vs. MIX) and concentrations of whole blood Se were adequate throughout the duration of this study (Figure 2, [39,40]). During the depletion period, whole blood concentrations of Se declined for both treatment groups, and subsequently increased during the period of repletion. On each treatment, the ISe supplemented cows had a numerically greater concentration of peripheral Se compared to the MIX treatment group. However, within each group, the levels remained relatively stable until the experimental endpoint. There was no effect (*p* > 0.05) of form of Se at any time point during the study. There was an effect of time (*p* < 0.0001), but there was no treatment X time interaction (*p* > 0.1).

### 3.2. Real-Time RT-PCR Analysis of Selenoprotein and Receptor mRNA Transcripts

**Figure 3:**
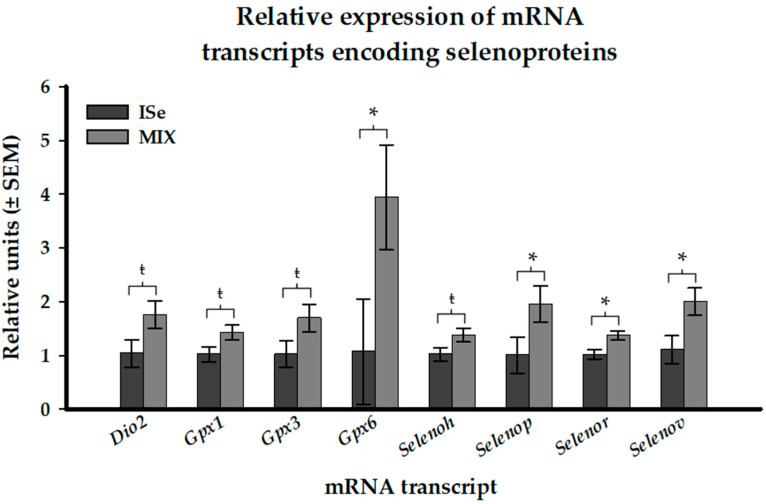
Effect of form of Se on the expression of mRNA transcripts encoding selenoproteins in the CL of cows supplemented with vitamin mineral mixes containing Se as ISe (sodium selenite; *n* = 5) or a 1:1 combination (MIX) of ISe and OSe (Sel-Plex; *n* = 5). *p*-values are associated with Student’s *t*-Test. Significant differences at *p* < 0.05 are indicated by an asterisk and tendencies at 0.05 ≤ *p* < 0.1 are indicated by ŧ.

Twenty-six transcripts encoding selenoproteins and three transcripts encoding receptors for selenoprotein P were targeted via qPCR analysis. The MIX form of supplemental Se significantly (*p* < 0.05) increased the abundance of four key transcripts encoding *Gpx6*, *Selenor*, *Selenov*, and *Tfrc* in the CL (Figure 3 and Table 1). Additionally, the relative abundance of *Dio2*, *Gpx1*, *Gpx3*, *Selenoh*, and *Selenop* tended (0.05 ≤ *p* < 0.1) to be increased in the MIX treatment group. The relative level of expression of the other targeted selenoprotein transcripts or selenoprotein P receptors was not affected by treatment (Table 1).

### 3.3. Concentrations of P4, Luteal Weight, and Luteal Diameter

The concentration of P4 was quantified in serum collected on Days 5, 6 and 7 of the estrous cycle. Although in the present study there was no observed effect of form of Se on systemic P4 (Table 2, Year 3), the difference of ~1 ng/mL between the treatment groups on Day 7 is consistent with previous studies from our lab [33,34] that used a larger number of animals (Table 2, Year 1 and Year 2). Additionally, there was no difference (*p* > 0.05) in the weight or the diameter of the CL, consistent with our previously reported results [33].

### 3.4. Production of Progesterone In Vitro

Luteal production of P4 was determined in vitro by treating dissociated luteal cells with or without a low or high concentration of three agonists, PGE_2_, LH, or hCG, for 96 h. Culture media was collected every 24 h for quantification of P4. Across all dietary and agonist treatments, there was a significant effect of time of culture (*p* < 0.0001) as concentrations of P4 in the culture media decreased with each subsequent 24-h period. There was a significant interaction (*p* < 0.0001) between time in culture and agonist treatment. There was no significant three-way interaction (*p* > 0.05) of dietary treatment, agonist treatment, and time in culture.

**Figure 4:**
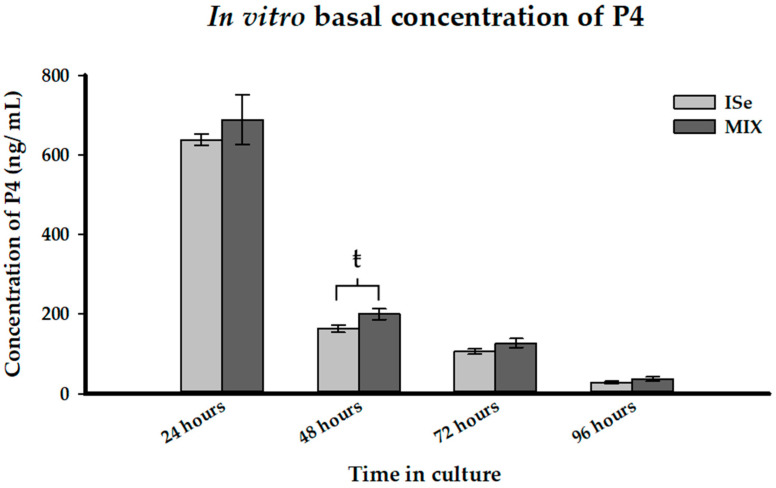
Effect of treatment (form of Se) on basal media concentrations of P4 (ng/mL; LS mean ± SEM) collected from in vitro culture every 24 h for 96 h from cows supplemented with either sodium selenite ISe (sodium selenite; *n* = 5) or a 1:1 combination (MIX) of ISe and OSe (Sel-Plex; *n* = 5) treatments. Data were analyzed using a split plot design for repeated measure. Significant differences were determined at *p* < 0.05 and tendencies were determined at 0.05 ≤ *p* < 0.1. Tendencies are indicated by ŧ.

In basal, untreated cultures, there was no significant difference (*p* > 0.1) between dietary treatment groups in the production of P4; however, there tended (199.5 ± 13.9 vs. 163.0 ± 8.9, *p* = 0.07) to be a greater concentration of P4 in the media collected at 48 h from CL retrieved from MIX-treated cows compared to ISe-treated cows (Figure 4).

**Figure 5:**
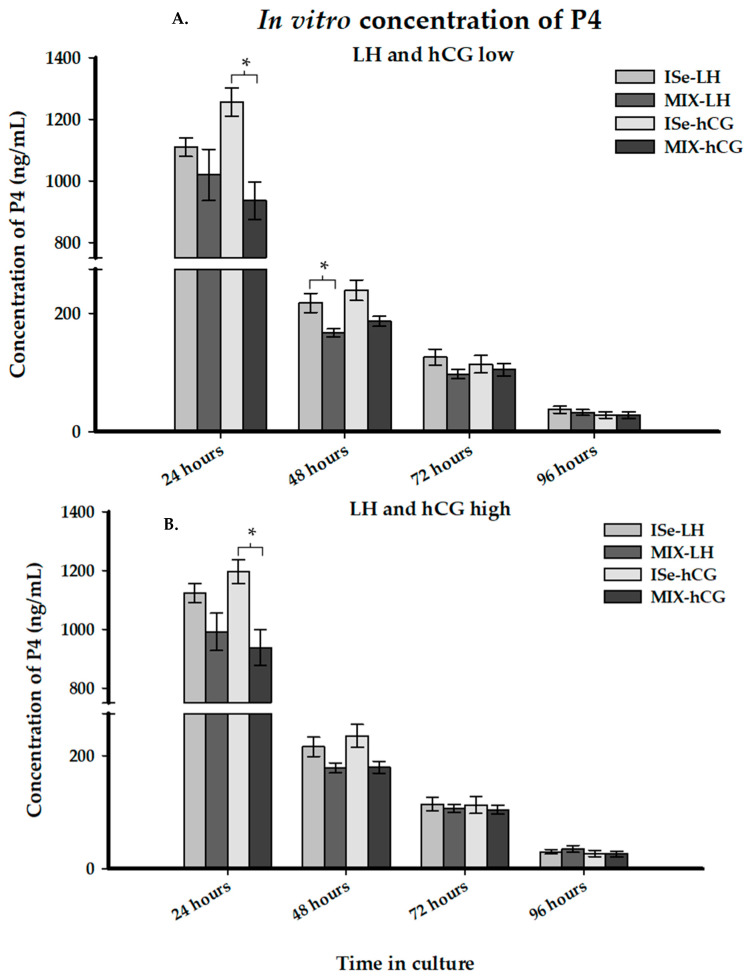
Effect of form of Se on concentrations of P4 in culture media (ng/mL; LS mean ± standard error of the mean) collected after in vitro culture of dissociated luteal cells with a low (**A**) or high (**B**) dose of LH (1 or 10 IU/mL) or hCG (10 or 50 ng/mL). Corpora lutea were recovered from cows supplemented with either ISe (Sodium selenite; *n* = 5) or a 1:1 combination (MIX) of ISe and OSe (Sel-Plex; *n* = 5) on Day 7 post-estrus. Data were analyzed using split plot design for repeated measure. Significant differences were determined at *p* < 0.05 and are designated by an asterisk.

After 24 h of culture, both the low and high doses of hCG increased the production of P4 in luteal cells collected from cows supplemented with ISe compared to MIX (*p* < 0.05, Figure 5A,B), an unexpected result. There was no other significant difference at 24 h in vitro (*p* > 0.1). After 48 h of culture, the low dose of LH also increased the concentration of P4 in luteal cultures from ISe- compared to MIX-treated cows (*p* < 0.05). At 72 and 96 h of culture, there were no differences between dietary treatments in the production of P4 in response to LH or hCG. Furthermore, there was no effect of form of Se on PGE_2_-treated luteal cells throughout the 96 h of culture (*p* > 0.05, data not shown).

### 3.5. Real-Time PCR Analysis of Steroidogenic and Cholesterol Related mRNA Transcripts

**Figure 6:**
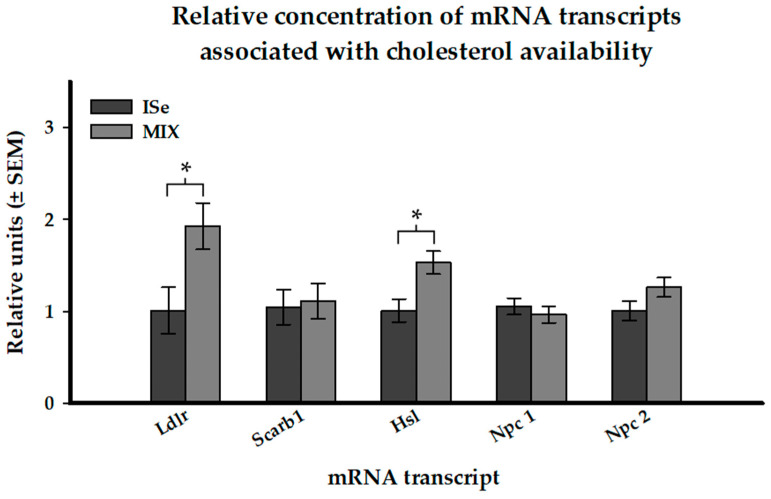
Effect of form of Se on the expression of mRNA transcripts associated with the availability of cholesterol in the CL of cows supplemented with Se in vitamin mineral mixes as ISe (sodium selenite; *n* = 5) or a 1:1 combination (MIX) of ISe and OSe (Sel-Plex; *n* = 5). *p*-values are associated with Student’s *t*-test. Significant differences at *p* < 0.05 are indicated by an asterisk.

The relative abundance of 21 mRNA transcripts associated with P4 biosynthesis and regulation were analyzed via qPCR. Form of Se did not (*p* > 0.05) affect the abundance of the five targeted enzymatic transcripts (Table 3). Of the targeted receptor transcripts, the expression of mRNA encoding the nuclear P4 receptor (*Pgr*) was decreased in CL retrieved from MIX versus ISe treated cows (*p* < 0.05). Of the five targeted transcripts associated with cholesterol availability, the level of expression of mRNA encoding both *Ldlr,* and *Hsl* was increased in CL retrieved from MIX versus ISe supplemented cows (Figure 6, *p* < 0.05).

## 4. Discussion

In this study, we sought to investigate the mechanism responsible for increases in early luteal phase concentrations of peripheral P4 after supplementation with MIX versus the industry standard ISe [33,34]. The specified objectives were to investigate the effect of supplementation with ISe or MIX-form Se on (1) the relative abundance of mRNA transcripts that encode selenoproteins and targeted steroidogenic enzymes in the CL, and (2) the ability of dissociated luteal cells to synthesize P4 in response to key agonists in vitro. We first verified our model by demonstrating the diets containing distinct Se-form specific vitamin-mineral mixes resulted in a selenium-adequate status in all animals during experimentation [24,39,40]. Further, we analyzed serum concentrations of P4 collected from cows on Days 5 to 7 of the early luteal phase to model the previously defined MIX-induced increase in P4. Our previous studies reported a 1.7 ng/mL increase in Day 6 (year one) concentrations of P4 [33], and a 1.0 ng/mL increase in Day 7 (year 2) P4 [34]. In the present study, we mimicked this difference with a 1.1 ng/mL increase in P4 on day 7 of the estrous cycle in the MIX treatment group. Although this difference was not statistically significant, we suspect that the lack of an effect in the serum concentration of P4 may be due to (1) the smaller number of animals in this more intensive study, and/or (2) a possible dietary interaction because our previous studies were performed using cattle maintained on toxic endophyte-infected tall fescue pastures, versus the study herein where cattle grazed novel, nontoxic endophyte-infected tall fescue pastures, followed by a silage-based diet over the winter months of January to April. Importantly, in both Cerny, et al. [33] and the current study, this increase in peripheral concentrations of P4 occurred in the absence of any treatment effect on weight or diameter of the CL, suggesting increased capacity of individual steroidogenic cells to synthesize P4.

Regarding selenoproteins, the physiological incorporation of Se into any tissue is dependent upon the form available [6]. SeMet enters the methionine metabolic pool and readily competes with Met for incorporation into protein by tRNA. The amount of incorporation of SeMet compared to methionine is dependent on their relative concentrations [56]. Alternatively, SeMet can be converted to SeCys via the intermediate selenocystathionine and the enzymatic activity of both cystathionine B-synthase and cystathionine [6,57]. The synthesized SeCys or dietary SeCys is transformed into selenide by β-lyase activity [6]. Contrary to the organic forms of Se, once consumed, selenate is easily converted to selenite and then further converted to selenide [57]. The formation of selenide is where the metabolic pathways for organic and inorganic forms of Se converge. Selenide is converted to the intermediate selenophosphate by the known selenoprotein enzymes selenophosphate synthetases (SEPHS1 and SEPHS2) [58,59]. Selenophosphate then interacts with a charged serine present on the specific ^SeCys^tRNA, followed by the conversion of Ser-^SeCys^tRNA to SeCys-^SeCys^tRNA for incorporation into selenoproteins [6,60].

In the CL, ROS including hydrogen peroxide and hydroxyl radicals are produced as a byproduct of aerobic metabolism when the P450/450 system catalyzes the reaction to produce P4 [61,62]. Similarly to what occurs in other cell types, ROS are also generated in the luteal cells during the production of ATP from oxidative phosphorylation by the oxidases NADH and NADPH, and by the activity of xanthine oxidase [63,64]. Therefore, antioxidants must be active to regulate the concentration of ROS and maintain cellular viability, as the concentration of ROS and antioxidants are closely related to luteal function [65,66,67,68,69]. Intra-luteal concentrations of ROS can be inhibitory to the production of P4 during luteolysis and pregnancy in rats [70,71]. Hence, maintenance of cellular function and production of P4 requires the regulation of concentrations of ROS by antioxidants. Unregulated ROS or increases in intracellular concentrations of ROS have been associated with apoptosis of the steroidogenic cells and luteolysis of the CL [72,73,74,75].

GPX variants are known to reduce hydrogen peroxide [9] and relevant to the current study, we observed a significant increase in the abundance of mRNA encoding *Gpx6* and a tendency of increasing *Gpx1* and *Gpx3* mRNA content in the CL from MIX supplemented cows. The most abundant glutathione peroxidase is the cytosolic GPX1. Vu, et al. [72] demonstrated that GPX1 protein abundance and activity is greatest in the early and mid-luteal stages aligning with the increases in the production of P4 at this time. In contrast, we did not observe any difference in the mitochondrial GPX4 which has the ability to alleviate apoptogenic protein release on the inner membrane of the mitochondria [76]. The roles of the GPX3 and GPX6 are not clearly defined in the CL, although we speculate that they exert a similar function related to reducing ROS. GPX3 is most highly abundant in blood [77], and previously researchers have demonstrated that the mRNA encoding *Gpx6* has been located in limited tissues including embryos, olfactory epithelium, sperm, seminal plasma, and the ovaries in humans [77,78,79,80,81,82]. The present study provides novel evidence of the presence and role for GPX6 in the CL of cows, and that the *Gpx6* mRNA abundance is significantly affected by the form of Se, with mRNA encoding *Gpx6* being increased in the CL from cows in the MIX treatment group. Other selenoproteins with antioxidant capabilities, and in which we observed a significant effect or tendency to increase abundance in the MIX treatment group of the respective mRNA transcripts encoding *Selenop* [14], *Selenoh* [16], and *Selenor* [19]. Of note, SELENOP has redox capabilities; however, its main physiological function is to maintain homeostasis of the concentration of Se [83]. It is primarily synthesized in the liver and transported through the plasma to target tissues to be internalized in the cell by receptor-mediated endocytosis [83]. Hence, it is of interest that the mRNA encoding *Selenop* was significantly upregulated in MIX treated cows, although the relevance within the CL is unclear at this time.

Of equal importance to the current study, systemic P4 is affected by a plethora of factors, including the rate of P4 synthesis in the CL, the rate of catabolism in the liver, and various luteotropic and luteolytic hormones [84,85,86,87,88,89]. In the cow, LH is considered the primary luteotropic hormone. In vitro stimulation of luteal cells with LH can increase the production of P4 by up to 20-fold [85,86]. The small steroidogenic cells of the CL contain receptors for LH (luteinizing hormone G-protein coupled receptor, LHCGR) which can assert its effects by acting through the protein kinase A (PKA) second messenger system to stimulate production of P4 [85,86]. However, large steroidogenic luteal cells that contribute a 20-fold greater amount of the P4 per cell are devoid of the LH receptor; thus, they do not respond to simulation by that gonadotropic hormone [86,90]. In the present study, we unexpectedly observed minor ISe-induced increases in P4 after treatment of luteal cells with LH or hCG in vitro, with hCG stimulating the small luteal cell similarly to LH [91]. Considering the relative level of expression of mRNA encoding the LH/hCG receptor (1.05 vs. 0.69 ± 0.14, ISe vs. MIX, relative units ± SEM, *p* = 0.11) that we observed in luteal tissue herein, a form of Se-induced effect on small luteal cell steroidogenesis may be apparent.

Large luteal cells contain receptors, including those for PGE_2_ and PGF_2α_ [86,87]. PGE_2_ has a luteotropic and luteoprotective role in the CL and PGF_2α_ is luteolytic [86,87]. In ruminants, treating luteal cells with PGE_2_ has resulted in an increase in the production of P4 [92,93,94], increased protection of the CL from luteolysis [95,96,97,98], and stimulation of the secretion of P4 by binding to the prostaglandin E receptors 1-4 (EP1, EP2, EP3, and EP4) on the plasma membrane and activation of the cAMP-mediated signaling pathway [88,89]. We did not observe an effect of treatment with PGE_2_ on luteal cell-production of P4 in the study, nor an effect of form of Se on the expression of mRNA encoding the *Ep1-4* receptors.

Intuitively, there is no clear connection between feeding a MIX Se-form supplement and the observed increases in systemic concentrations of P4. Therefore, to investigate this mechanism, we first analyzed for differences in the expression of transcripts for key steroidogenic enzymes and associated receptors. We hypothesized that there would be an increase in enzymatic transcripts and receptor transcripts that favor the production of P4. Our initial hypothesis was not substantiated. Interestingly, among the transcripts analyzed, the content of mRNA encoding the nuclear *Pgr* but not the membrane components *Pgrmc1* and *Pgrmc2* was decreased in CL from MIX-supplemented cows suggesting down-regulation of PGR mediated events in those animals. Both on and within steroidogenic luteal cells, P4 auto-regulates the further production of P4 by binding to either the nuclear membrane receptors (PGR-A and PGR-B), or the cytoplasmic and endoplasmic membrane bound P4 receptors, P4 receptor membrane components 1 and 2 (PGRMC1 and PGRMC2, respectively). P4 also binds the progestin and adipoQ receptor family members 5, 7, and 8 (PAQR5, PAQR7, and PAQR8, respectively) [99,100].

**Figure 7:**
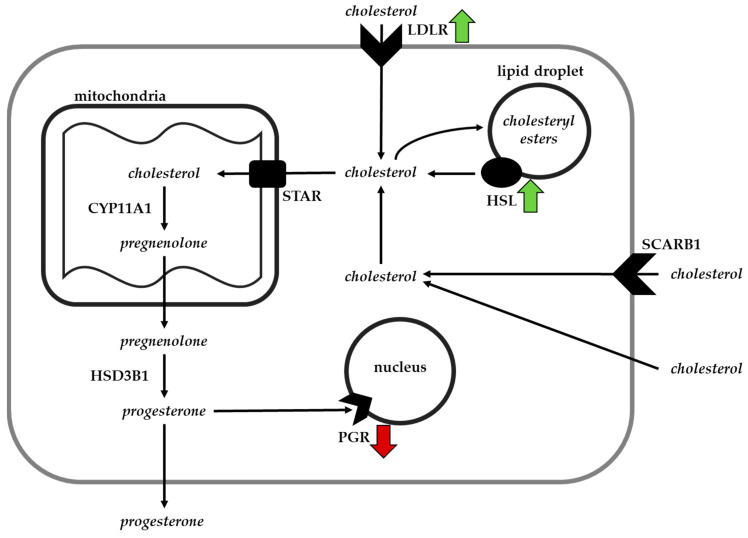
Mechanism of the availability of cholesterol and steroidogenesis in a steroidogenic luteal cell, emphasizing the four potential sources of cholesterol that are available as a substrate. Each arrow represents a significant difference in the abundance of mRNA associated with each respective protein. The arrow pointing upward or downward indicates that the mRNA is significantly upregulated (green) or downregulated (red) respectively in the MIX treatment group compared to the ISe treatment group.

After analyzing the results of transcripts related to the production of P4, concurrent with the results from our in vitro study, it was clear that the regulatory activity of the peripheral production of P4 had to be either upstream or downstream of the targeted steroidogenic pathway (Figure 7). Mechanistically, the primary substrate for the production of P4 is cholesterol, which is made available to steroidogenic luteal cells via four mechanisms: conversion of low-density lipoproteins (LDL), uptake of high-density lipoproteins (HDL), the uptake of free cholesterol, or de novo synthesis [101,102,103]. The conversion of LDL and HDL into free cholesterol is the prominent pathway to make this substrate available for the production of P4 in the CL [104,105,106]. The uptake of LDL occurs by binding to the LDL receptor (LDLR) and triggering receptor mediated endocytosis [107]. The endosome containing the LDL combines with lysosomes and LDL is dissociated from the receptor to be broken down into free cholesterol [108]. Transport out of the lysosome occurs by binding the cholesterol transporters: Niemann-Pick C1 protein (NPC1) and Niemann-Pick C2 protein [NPC2 [109,110,111]]. LDL-derived free cholesterol is rapidly exported out of the lysosomal compartments via the actions of NPC1 and NPC2, and without these transporters, cholesterol and other lipids would accrete within the lysosomes [112]. In contrast, HDL is utilized as a substrate for cholesterol by first binding to the scavenger receptor class B type 1 (SCARB1) on the cell surface. The lipoprotein is not entirely internalized; rather, the cholesteryl esters are selectively delivered into the cell to be hydrolyzed into free cholesterol [113,114,115]. Another mechanism relevant to the present study and associated with the availability of P4 in steroidogenic cells is the hydrolysis of cholesteryl esters to free cholesterol from lipid droplets. This reaction is stimulated by hormone-sensitive lipase (HSL) which results in free cholesterol and fatty acids that can then act as precursors for various physiological functions, including the production of steroid hormones and cellular energy [116,117,118,119,120].

Once cholesterol is free, it is transported into the mitochondria by binding the steroidogenic acute regulatory protein (STAR), which then interactions with membrane proteins on both the outer and inner mitochondrial membranes to facilitate the transport into the inner mitochondrial matrix [121]. This mechanism mediates the rate at which P4 can be produced [122,123,124]. Cholesterol is then converted into pregnenolone via the actions of cytochrome P450 family 11 member1 (CYP11A1) located on the inner mitochondrial membrane [125,126,127,128]. Once transported out of the mitochondria, the enzymes hydroxy-delta-5-steroid dehydrogenase, 3 beta- and steroid delta isomerase 1 (HSD3B1) located in the endoplasmic reticulum further converts pregnenolone into P4 [129] allowing autocrine, paracrine, and endocrine activity of the hormone.

We targeted five transcripts (*Ldl*, *Scarb1*, *Npc1*, *Npc2*, and *Hsl*) that are indicative of the availability of cholesterol to steroidogenic cells. Of these, transcripts encoding *Ldlr* and *Hsl* were significantly upregulated in the MIX Se treatment group suggesting that the MIX-induced increase in in vivo P4 is due, in part, to stimulation of cholesterol uptake. Since we observed increases in mRNA encoding the *Ldlr* transcript in the MIX-supplemented Day 7 CL, the failure to observe a MIX-induced increase in the production of P4 in vitro appears to be due to the fact that there is limited cholesterol available in the serum-free media for the cells to internalize as substrate. Thus, these cells cannot recapitulate the in vivo increase in the production of P4 by the CL from cows supplemented with the MIX form of Se.

Given this, the mechanism between dietary form of Se and the MIX-induced increase in early luteal phase P4 requires further research to define. However, it appears that it could partially be due to differences in the physiological integration of both selenoproteins and cholesterol, with cholesterol being the primary substrate for the production of P4 by the CL. Selenium is an integral component of GPX enzymes, which have antioxidant activity by removing hydrogen peroxide and protecting against the aggregation of reactive oxygen species (ROS). We have previously demonstrated that feeding the MIX form or the organic form of Se significantly upregulates the *Gpx4* transcript in the pituitary of steers when compared to ISe [22], which asserts antioxidant properties there. ROS are generated in the mitochondria of the CL and have been shown to reduce the production of P4 [71,130,131,132,133]. Additionally, ROS damage LH receptors [133,134], inhibit the transfer of cholesterol to the mitochondria for the synthesis of P4 [131], and inhibit the enzymatic activity of P450scc [132]. In concert, the information discussed herein provides a plausible mechanism for the association between supplementing the MIX Se-form and increased production of P4. However, additional research that analyzes transcripts associated with cholesterol biosynthesis and the production of ROS within the CL, are warranted to further define the interrelationships between the form of dietary Se, systemic concentrations of cholesterol, and peripheral concentrations of P4.

## 5. Conclusions

In this study, we sought to quantify Se form-induced changes in the expression of mRNA encoding selenoproteins and to investigate the mechanism(s) responsible for the previously reported increase in early luteal phase concentrations of P4 in cows supplemented with the MIX form of Se. Though the mechanism is still not completely defined, we have demonstrated that the form of selenium does not affect steroidogenic enzyme mRNA expression but does alter mRNA transcripts associated with the availability of cholesterol to steroidogenic luteal cells. Further research is necessary to determine how the bioavailability of cholesterol is affected. Overall, it appears that the MIX-induced increase in early luteal P4 is not directly mediated by an increase in the key steroidogenic transcripts but by an increase in cholesterol uptake, through at least the LDLR. Understanding the mechanism of the MIX-induced increase in P4 is requisite, as this novel dietary approach offers a producer-friendly avenue to increase fertility outcomes in beef cows grazing forages in areas with soils deficient in Se.

## Figures and Tables

**Table 1:** Relative abundance of mRNA encoding selenoproteins and selenoprotein P receptors in the CL of cows supplemented with vitamin-mineral mixes containing Se as sodium selenite (ISe, *n* = 5) or a 1:1 equimolar mix of ISe and OSe (MIX, SEL-PLEX, *n* = 5) ^1^.

Gene	Gene Name	qPCR			
		ISe	MIX	SEM	*p*-Value ^2^
Iodothyronine deiodinases					
*DIO1*	Iodothyronine deiodinase 1	1.6668	1.3540	0.5915	0.89
*DIO3*	Iodothyronine deiodinase 3	1.8881	0.7281	0.5667	0.54
Glutathione peroxidases					
*GPX2*	Glutathione peroxidase 2	1.1133	1.3412	0.2026	0.45
*GPX4*	Glutathione peroxidase 4	1.0200	0.8963	0.0906	0.36
Thioredoxin reductases					
*TXNRD1*	Thioredoxin reductase 1	1.0338	1.2380	0.1528	0.37
*TXNRD2*	Thioredoxin reductase 2	1.0034	1.0816	0.0435	0.24
*TXNRD3*	Thioredoxin reductase 3	1.0357	1.2130	0.1613	0.50
Other selenoproteins					
*SELENOF*	Selenoprotein F	1.0223	0.8537	0.0983	0.26
*SELENOI*	Selenoprotein I	1.0089	1.2904	0.1215	0.14
*SELNOK*	Selenoprotein K	1.0143	1.2131	0.0810	0.12
*SELNOM*	Selenoprotein M	4.6687	7.5038	2.9960	0.52
*SELENON*	Selenoprotein N	1.0196	1.0072	0.0733	0.91
*SELENOO*	Selenoprotein O	1.0234	1.1227	0.0808	0.33
*SELENOS*	Selenoprotein S	1.0131	1.2860	0.1229	0.16
*SELENOT*	Selenoprotein T	1.0398	0.9553	0.1090	0.60
*SELENOW*	Selenoprotein W	1.0183	1.1465	0.1134	0.44
*SEPHS1*	Selenophosphate synthetase 1	1.0655	1.6289	0.2718	0.18
*SEPHS2*	Selenophosphate synthetase 2	1.0142	1.0127	0.0738	0.99
Selenoprotein P receptors					
*LRP2*	LDL receptor related protein 2	1.0335	1.2207	0.1478	0.40
*LRP8*	LDL receptor related protein 8	1.0647	1.2923	0.1858	0.41
*TFRC* ^†^	Transferrin receptor	1.0104 ^a^	1.5173 ^b^	0.1116	0.01

^1^ Se was supplemented at 35 ppm as either inorganic (ISe; sodium selenite), or a 1:1 combination (MIX) of ISe and OSe (SEL-PLEX). ^2^ *p*-values are associated with Student’s *t*-Test. ^†^ Means with different superscripted letters differ at *p* < 0.05.

**Table 2:** Effect of treatment (form of supplemental Se) on the concentration of systemic P4 in cows between days 4 and 10 of the estrous cycle. Cows were supplemented with Se as sodium selenite (ISe, *n* = 5) or a 1:1 equimolar mix of ISe and OSe (MIX, SEL-PLEX, *n* = 5) ^1,†^.

	Treatment		
Variable	ISe LS Mean ± SEM	MIX LS Mean ± SEM	*p*-Value ^2^
Progesterone (ng/mL) Year 1 *			
No. of cows (*n*)	9	9	
Day 6 ^†^	3.44 ± 0.18 ^a^	5.14 ± 0.60 ^b^	0.035
Year 2 **			
No. of cows (*n*)	12	12	
Day 4	1.02 ± 0.22	0.94 ± 0.12	0.740
Day 7 ^†^	2.92 ± 0.27 ^a^	3.91 ± 0.16 ^b^	0.006
Day 10	7.17 ± 0.54	6.36 ± 0.55	0.308
Year 3			
No. of cows (*n*)	5	5	
Day 5	0.59 ± 0.58	1.20 ± 0.55	0.456
Day 6	0.86 ± 0.55	1.19 ± 0.55	0.678
Day 7	1.87 ± 0.55	2.92 ± 0.55	0.198
CL weight (g)	6.07 ± 0.82	6.77 ± 0.82	0.563
CL diameter (mm)	22.3 ± 1.09	23.2 ± 1.09	0.576

^1^ Selenium was supplemented at 35 ppm as either inorganic (ISe; sodium selenite), or a 1:1 combination (MIX) of ISe and OSe (SEL-PLEX). Selenium was supplemented individually using in-pasture Calan gates [26]. ^2^ *p*-values associated with one-way ANOVA (year 1, OSe treatment not shown), ANOVA with repeated measures (year 2 and year 3), and Student’s *t*-Test (CL weight and CL diameter). ^†^ Means with different superscripted letters differ at *p* < 0.05. * Reported in [33]. ** Reported in [34].

**Table 3:** Effect of form of Se on the relative expression of mRNA transcripts encoding steroidogenic enzymes and receptors in the Day 7 CL. Cows were supplemented with Se as sodium selenite (ISe, *n* = 5) or a 1:1 equimolar mix of ISe and OSe (MIX, SEL-PLEX, *n* = 5) ^1,†^.

Gene	Gene Name	qPCR			
		ISe	MIX	SEM	*p*-Value ^2^
Enzymatic transcripts					
*STAR*	Steroidogenic acute regulatory protein	1.0231	1.2259	0.1132	0.2410
*CYP11A1*	Cytochrome P450, family 11, subfamily A, polypeptide 1	1.0068	0.9822	0.0503	0.7371
*HSD3B1*	Hydroxy-delta-5-steroid dehydrogenase, 3 beta- and steroid delta-isomerase 1	1.0014	0.9521	0.0398	0.4069
*PTGS2*	Prostaglandin-endoperoxide synthase 2 (COX2)	1.0079	0.9396	0.1574	0.7668
*PTGES*	Prostaglandin E synthase	1.4308	0.9376	0.3539	0.3533
Receptor transcripts					
*LHCGR*	Luteinizing hormone (LH) G-protein coupled receptor	1.0484	0.6859	0.1433	0.1112
*PGR*	Nuclear progesterone receptor	1.0076 ^a^	0.8415 ^b^	0.0508	0.0495
*PGRMC1*	Progesterone receptor membrane component 1	1.0355	0.9044	0.1060	0.4071
*PGRMC2*	Progesterone receptor membrane component 2	1.0248	1.0553	0.0970	0.8295
*EP1*	Prostaglandin E receptor 1	1.2310	1.4844	0.0721	0.5832
*EP2*	Prostaglandin E receptor 2	1.0385	1.0890	0.1392	0.8041
*EP3*	Prostaglandin E receptor 3	1.0286	0.7820	0.1045	0.1337
*EP4*	Prostaglandin E receptor 4	1.0313	1.2515	0.1524	0.3368
*PAQR5*	Progestin and adipoQ receptor family member 5 (mPR γ)	1.0342	0.9295	0.1187	0.5502
*PAQR7*	Progestin and adipoQ receptor family member 7 (mPR α)	1.0165	0.8922	0.0804	0.3058
*PAQR8*	Progestin and adipoQ receptor family member 8 (mPR β)	1.0042	1.0206	0.0678	0.8687
*PGTFR*	Prostaglandin F receptor	1.0493	0.8624	0.1735	0.4680

^1^ Selenium was supplemented at 35 ppm as either inorganic (ISe; sodium selenite), or a 1:1 combination (MIX) of ISe and OSe (SEL-PLEX). Selenium was supplemented individually using in-pasture Calan gates [26]. ^2^ *p*-values associated with Student’s *t*-Test. ^†^ Means with different superscripted letters differ (*p* < 0.05).

## Data Availability

Not applicable.

## Supplementary Materials

The following are available online at https://www.mdpi.com/article/10.3390/ani12030313/s1, Table S1: Primer sets and product identities of qPCR analysis of selenoprotein-associated genes, Table S2: Primer sets and product identities of qPCR analysis of reference and steroidogenesis-associated genes.
