# Supplementary material for: Form of Supplemental Selenium Affects the Expression of mRNA Transcripts Encoding Selenoproteins, and Proteins Regulating Cholesterol Uptake, in the Corpus Luteum of Grazing Beef Cows
Source: Animals (Basel). 2022 Jan 27;12(3):313. doi: 10.3390/ani12030313 (PMC8833813; doi:10.3390/ani12030313)
Supplement: Supplementary file 1 [file animals-12-00313-s001.zip › animals-1484171-supplementary.pdf]

Supplementary Materials

Table S1. Primer sets and product identities of qPCR analysis of selenoprotein-associated genes.

Gene	Gene Name	Accession Number[1]	Oligonucleotide Primer Design (5′ to 3′) direction	Amplicon length (bp)	Product identity [2]
Enzymatic transcripts					
DIO1	Iodothyronine deiodinase 1	NM_001122593.2	F: TCCTGTAGTCCGCCTGTCA<br>R: TCCGGTGATTCTTGATGTCCA	242	99%
DIO2	Iodothyronine deiodinase 2	NM_001010992.4	F: GATGGGCATCCTCAGCGTAG<br>R: TTCTCCTGGGCACCATTTCC	315	100%
DIO3	Iodothyronine deiodinase 3	NM_001010993.3	F: AAGTGGAGCTCAACAGCGAT<br>R: AGTCGAGGATGTGCTGGTTC	213	100%
Glutathione peroxidases					
GPX1	Glutathione peroxidase 1	NM_174076.3	F: GCAACCAGTTTGGGCATCAG<br>R: TAGGGTCGGTCATGAGAGCA	210	100%
GPX2	Glutathione peroxidase 2	NM_001163139.2	F: AACAGCCTCAAGTACGTCCG<br>R: TCGGTCATGAGGGAAAACGG	158	100%
GPX3	Glutathione peroxidase 3	NM_174077.5	F: GCACCATCTATGAGTACGGGG<br>R: CCCCATTCACATCGCCTTTC	315	100%
GPX4	Glutathione peroxidase 4	NM_174770.3	F: GATCAAAGAGTTCGCCGCTG<br>R: CCATACCGCTTCACCACACA	198	100%
GPX6	Glutathione peroxidase 6	NM_001163142.1	F: CACTGTTCCTGGTCGGCTTA<br>R: CCCAGCACAACTACACCGAA	259	100%
Thioredoxin reductases					
TXNRD1	Thioredoxin reductase 1	NM_174625.5	F: AAGGCCGCGTTATTTGGGTA<br>R: CCTGGTGTCCCTGCTTCAAT	306	100%
TXNRD2	Thioredoxin reductase 2	NM_174626.2	F: CAAATGGCTTCGCTGGTCAC<br>R: TTCGTATGCACACCAGCCTT	230	100%

TXNRD3	Thioredoxin reductase 3	XM_015468824.1	F: CGGCGTATGACTACGACCTC<br>R: GACTGTACTCCCAGCCGAAC	249	100%
Other selenoproteins					
SELENOF	Selenoprotein F	NM_001034759.2	F: GCAGCTCCTGTGATTTGCTT<br>R: TTTAGCACAGGGTCTGAACCG	241	100%
SELENOH	Selenoprotein H	NM_001321327.1	F: CACGAGCTGACGAGTCTACG<br>R: CTTCTTCAGCTCCTCCAGCA	235	100%
SELENOI	Selenoprotein I	NM_001075257.2	F: TCTGGCTTTCTGCTGGTTGT<br>R: TGGTCAAAAAGCTCCCCCAG	212	100%
SELENOK	Selenoprotein K	NM_001037489.3	F: CCGTTTTGTCGATTCACGGC<br>R: CAGATGAGCTTCCGTAGCCT	278	100%
SELENOM	Selenoprotein M	NM_001163171.2	F: CCCACTCTACCACAACCTGG<br>R: ACCTAAAGGTCTGCGTGGTC	249	100%
SELENON	Selenoprotein N	NM_001114976.2	F: GTGGCCATGTACCCCTTCAA<br>R: GGGATGGGTTCTCCTGGTTG	265	100%
SELENOO	Selenoprotein O	NM_001163193.2	F: TGGACAGGTATGACCCCGAT<br>R: ATCTTCTGCAGGTAGTGCCG	202	100%
SELENOP	Selenoprotein P	NM_174459.3	F: TCAGGTCTTCATCACCACCA<br>R: GTGGCAACAGCAGCTACTCA	201	100%
SELENOR	Selenoprotein R, Methionine sulfoxide reductase B1	NM_001034810.2	F: GAACCACTTTGAGCCGGGTA<br>R: GGCCATCGTTCAGGAACTCA	221	100%
SELENOS	Selenoprotein S	NM_001046114.3	F: CCCACCCTCGAGACCGA<br>R: GCCCAGGACTGTCTTCTTCC	394	100%
SELENOT	Selenoprotein T	NM_001103103.2	F: TGGTCACCTTCCATCCATGC<br>R: AAGAGGTACAACGAGCCTGC	240	100%
SELENOV	Selenoprotein V	NM_001163244.2	F: ACTCCATTGGCCACCGATTT<br>R: AGGCCACAGTAAACCACTCG	224	100%

SELENOW	Selenoprotein W	NM_001163225.1	F: AGTGTTCGTAGCGGGAAAGC<br>R: CGCGAGAACATCAGGGAAGG	233	98%
SEPHS1	Selenophosphate synthetase 1	NM_001075316.1	F: CAAAGCGAACCGGTGGATCT<br>R: GAGGTCACTGAGGACGTTGG	422	99%
SEPHS2	Selenophosphate synthetase 2	NM_001114732.2	F: GATCCCTACATGATGGGGCG<br>R: GTTTACCACCGTTTGCCCAC	219	100%
Selenoprotein P receptors					
LRP2	LDL receptor related protein 2	XM_024983502.1	F: GTGGTTTGGGTTACCGTTGC<br>R: GGCACCCTGTTAGCTGTGAT	304	99%
LRP8	LDL receptor related protein 8	NM_001097565.1	F: AGCCACCCTTTTGGGATAGC<br>R: AAGGCACAGGTACTCACAGC	231	100%
TFRC	Transferrin receptor	NM_001206577.1	F: CCAGGTTTAGTCTGGCTCGG<br>R: GGTCTGCCCAGAATATGCGA	339	99%

[1] These contents are associated with each gene symbol and are the accession numbers of the sequences retrieved from the NCBI RefSeq database for designing primers and probes.
[2] All qPCR products were validated by sequencing. The identity values (%) presented are the base pair ratios between the total amplicon length and the number of identical base pairs.

Table S2. Primer sets and product identities of qPCR analysis of reference and steroidogenesis-associated genes.

**Gene**	**Gene Name**	**Accession Number[1]**	**Oligonucleotide Primer Design (5′ to 3′) direction**	**Amplicon length (bp)**	**Product identity [2]**
Enzymatic transcripts					
STAR	Steroidogenic acute regulatory protein	NM_174189.3	F: CCCGAGACTTTGTGAGCGTA<br>R: GCGCAGGTGATTGGCAAAAT	275	99%
CYP11A1	Cytochrome P450, family 11, subfamily A, polypeptide 1	NM_176644.2	F: TTCAACCTCATCCTGACGCC<br>R: GTGCAAGAGGTGTGGACTGA	204	98%
HSD3B1	Hydroxy-delta-5-steroid dehydrogenase, 3 beta- and steroid delta-isomerase 1	NM_174343.3	F: GGTTCCTGGTGAGCGTTTCT<br>R: CAGCAGCTGGGTACCTTTCA	362	99%
PTGS2	Prostaglandin-endoperoxide synthase 2	NM_174445.2	F: CCCATGGGTGTGAAAGGGAG<br>R: TCCACCCCATGGTTCTTTCC	203	100%

PTGES	Prostaglandin E synthase	NM_174443.2	F: CGCTGCTGGTCATCAAAATGT<br>R: GGTCTCCATGTCATTCCGGT	173	97%
Receptor transcripts					
LHCGR	Luteinizing hormone (LH) G-protein coupled receptor	NM_174381.1	F: GCCTTTGACAACCTCCTCAAT<br>R: TCCAGGGAAATCAGCGTTGT	332	99%
PGR	Nuclear progesterone receptor	NM_001205356.1	F: CCCACAGGAGTTTGTGAAGC<br>R: AGTGCCCGGGACTGGATAAA	291	99%
PGRMC1	Progesterone receptor membrane component 1	NM_001075133.1	F: GGCCGTATGGAGTCTTTGCT<br>R: TTGTCTGAGTACACGGTGGG	217	100%
PGRMC2	Progesterone receptor membrane component 2	NM_001099060.1	F: GCTTGCGGTCAATGGGAAAG<br>R: GACGGTTCTTCCCCTGGTTT	264	99%
EP1	Prostaglandin E receptor 1	NM_001192148.1	F: GGCCGCTGTTTTTGGCCGTG<br>R: CCTCCATGGCTGCCCTTGGC	142	100%
EP2	Prostaglandin E receptor 2	NM_174588.2	F: GCTTCATCGGACACAAGCAG<br>R: CTCCGCCATGGATACCCTTT	197	100%
EP3	Prostaglandin E receptor 3	NM_181032.1	F: CGCCGTTGCTGATAATGATGT<br>R: GTCCTTTCAAAAGCTGGCAA	204	100%
EP4	Prostaglandin E receptor 4	NM_174589.2	F: CGGGACCAATGCATCATCCT<br>R: TTGGCCCTTCAAGTAGGTGG	241	100%
PAQR5	Progestin and adipoQ receptor family member 5 (mPR_{γ})	XM_024997926.1	F: GGTTCTTCTCGTGGAGGTTTGT<br>R: GTTCCTGGACATGGAGCTGAA	151	96%
PAQR7	Progestin and adipoQ receptor family member 7 (mPR_{α})	NM_001038553.1	F: CCGGCGGTCCATCTATGA<br>R: CCACCCCCTTCACTGAGTCTT	159	99%
PAQR8	Progestin and adipoQ receptor family member 8 (mPR_{β})	NM_001101135.2	F: TGTAGCCTTGCGAGACACAG<br>R: CAGCATCGCAGAAGAATGCC	214	100%
PTGFR	Prostaglandin F receptor	NM_181025.3	F: TGGTGTTCTCTGGTCTGTGC<br>R: GGCTAGGAGCCCCAGAAAAG	293	100%

Cholesterol related transcripts					
LDLR	Low density lipoprotein receptor	NM_001166530.1	F: CCCTGACTGCAAGGACAAGT<br>R: GGAGATGCACTCACCGCTTT	217	99%
SCARB1	Scavenger receptor class B member 1	NM_174597.2	F: CAGACATGGGCAACCTCTCT<br>R: TGGATGATCCCCTCAGGGTT	244	99%
HSL	Lipase E, hormone sensitive type	NM_001080220.1	F: GGGATATCTGAAGAGGCCTGG<br>R: GGCTGGTGCGAAAGAAGATG	362	99%
NPC1	NPC intracellular cholesterol transporter 1	NM_174758.2	F: GGTCATGAGCTGTGGCATCT<br>R: TAGTCCATGAGTGGCTCCCA	253	98%
NPC2	NPC intracellular cholesterol transporter 2	NM_173918.2	F: GGACTGCGGTTCTTGGGTC<br>R: GGGGCATCTGATTCCAGACT	226	99%
Housekeeping Transcripts					
ACTB	Actin beta	NM_173979.3	F: GAGCGGGAAATCGTCCGTGAC<br>R: GTGTTGGCGTAGAGGTCCTTGC	278	99%
HPRT1	Hypoxanthine phosphoribosyltransferase 1	NM_001034035.2	F: GCCAGCCGGCTACGTTAT<br>R: ATCCAACAGGTCGGCAAAGA	256	100%
SDHA	Succinate dehydrogenase complex flavoprotein subunit A	NM_174178.2	F: GCAGAACCTGATGCTTTGTG<br>R: CGTAGGAGAGCGTGTGCTT	185	99%

[1] These contents are associated with each gene symbol and are the accession numbers of the sequences retrieved from the NCBI RefSeq database for designing primers and probes.
[2] All qPCR products were validated by sequencing. The identity values (%) presented are the base pair ratios between the total amplicon length and the number of identical base pairs.
